# Single-trial visually evoked potentials predict both individual choice and market outcomes

**DOI:** 10.1038/s41598-023-41613-4

**Published:** 2023-09-01

**Authors:** John P. Veillette, Shannon L. M. Heald, Benjamin Wittenbrink, Katherine S. Reis, Howard C. Nusbaum

**Affiliations:** Department of Psychology, University of Chicago, Chicago, 60637 USA

**Keywords:** Decision, Human behaviour

## Abstract

A central assumption in the behavioral sciences is that choice behavior generalizes enough across individuals that measurements from a sampled group can predict the behavior of the population. Following from this assumption, the unit of behavioral sampling or measurement for most neuroimaging studies is the individual; however, cognitive neuroscience is increasingly acknowledging a dissociation between neural activity that predicts individual behavior and that which predicts the average or aggregate behavior of the population suggesting a greater importance of individual differences than is typically acknowledged. For instance, past work has demonstrated that some, but not all, of the neural activity observed during value-based decision-making is able to predict not just individual subjects’ choices but also the success of products on large, online marketplaces—even when those two behavioral outcomes deviate from one another—suggesting that some neural component processes of decision-making generalize to aggregate market responses more readily across individuals than others do. While the bulk of such research has highlighted affect-related neural responses (i.e. in the nucleus accumbens) as a better predictor of group-level behavior than frontal cortical activity associated with the integration of more idiosyncratic choice components, more recent evidence has implicated responses in visual cortical regions as strong predictors of group preference. Taken together, these findings suggest a role of neural responses during early perception in reinforcing choice consistency across individuals and raise fundamental scientific questions about the role sensory systems in value-based decision-making processes. We use a multivariate pattern analysis approach to show that single-trial visually evoked electroencephalographic (EEG) activity can predict individual choice throughout the post-stimulus epoch; however, a nominally sparser set of activity predicts the aggregate behavior of the population. These findings support an account in which a subset of the neural activity underlying individual choice processes can scale to predict behavioral consistency across people, even when the choice behavior of the sample does not match the aggregate behavior of the population.

## Introduction

It is both a widespread assumption and a common empirical finding that the choice behavior of individuals is related to the aggregate or consensus choice behavior of a group. Almost all research in the social and behavioral sciences relies on the notion of generalizing from small samples to the population. This notion justifies methodologies such as political polling, focus groups, A/B testing, and both small- and large-sample behavioral experiments. From this perspective, the population behavior is characterized by a central tendency, and deviations from that central tendency at the level of the individual are seen as idiosyncratic noise. The past few decades’ progress in neuroimaging, however, has enabled cognitive scientists to isolate component cognitive and neural processes that give rise to individual decisions. In this vein, researchers have begun to ask whether some such lower-level processes may generalize more readily from the individual to the population than others. In other words, is it possible to differentiate, for a single brain, between neural activity that predicts the population’s central tendency and that which only relates to the idiosyncrasies of an individual’s choice? Or phrased differently, can an individual’s brain activity predict aggregate behavior even when their own behavior does not? This line of research shifts the focus from “what is the central tendency of the population” to “what shared neural mechanisms make it such that a set of individuals can be said to come from the same population?” This distinction is critical in understanding the neural correlates of behavioral diversity and, conversely, the underpinnings of behaviorally consistency.

Recently, Knutson and Genevsky^1^ proposed a partial-scaling account of choice consistency, in which only a specific subset of the neural activity associated with an individual’s decision-making process is able to predict the aggregate behavior of a group. Building upon Samanez-Larkin and Knutson’s^2^ Affect-Integration model of value-based decision-making, they argue that initial affective responses to choice stimuli (e.g. “that’s a nice looking sports car”) would generalize well across people, thus reflecting both aggregate and individual behavior. In contrast, later integrative processes would be bogged down by more idiosyncratic and deliberative considerations (e.g. “I already own a car”). This proposal is supported by findings from Genevsky and colleagues’^3^ study, in which subjects were given a cash endowment and asked to make funding decisions about product pitches taken from a crowdfunding site Kickstarter. Affect-related fMRI BOLD responses in the nucleus accumbens predicted individual choices and, above-and-beyond subjects' behavioral responses, was able to predict whether the products would ultimately reach their funding threshold on Kickstarter. In contrast, medial prefrontal cortical activity implicated in later, integrative choice processes were only able to predict individual choice behavior. Importantly, these findings highlighted the utility of leveraging aggregate data from massive online marketplaces to shed light on neural processes measured in an individual.

Other models of decision-making, however, extend the neuroanatomy of decision-making processes outside of the traditional striatal-to-prefrontal circuit included in the Affect-Integration model. Increasing evidence from nonhuman animal models suggests that neuronal activity diverges along decision parameters during early sensory processing, perhaps reflecting a basic part of perceptual processes. Activity in early visual cortices was, until recently, thought to almost exclusively reflect ascending information coming from the optic nerve, with the magnitude of neural responses being modulated by attention. Such magnitude modulation in primate V4 (a “higher” visual cortex), however, has now been differentiated from attentional influence and characterized instead as a representation of expected reward; this reward representation modulates attentional responses downstream in the visual pathway instead of the other way around^4^. Activity correlated to timing of reward has been found as early in the visual pathway as V1 (primary visual cortex) in rats^5^. Thiele^6^ found that decision-related activity in the visual pathway of primates is spread roughly uniformly across the cortical hierarchy, challenging the notion that higher-level regions contribute more to decisions. Since these findings are based on perceptual discriminations in nonhuman animals, their applicability to human value-based decisions is largely unknown. However, such results dovetail with findings by Dmochowski et al.^7^ that inter-subject correlation (ISC) in scalp-recorded EEG predicts large-scale group preference for video stimuli, and fMRI covariation with that EEG ISC was found primarily in visual cortices. Those authors concluded that commonalities in sensory processing between people may partially account for inter-subject consistency in preferences; however, their continuous (rather than epoched) experimental design limited inferences about the time course of preference-related neural activity relative to the visual stimulus itself.

The implication that sensory cortices may be active in value-based decision processes raises a number of theoretic issues about the time-course of decision-related processing. For instance, when during visual processing does choice-predictive individual variation begin to emerge? Given that visual processing is generally assumed to be more consistent across subjects than, say, medial prefrontal cortical activity, decision-related mechanisms in the visual system are strong candidates for reinforcing choice consistency within a population. Do any of all visual system predictors of individual choice also predict group-aggregate outcomes, or again just some subset?

To investigate these questions, we used a multivariate pattern analysis approach, in which we trained a classifier to predict choice behavior from the single-trial EEG trace. Critically, this single-trial “neural decoding” approach allows us to assess how well our trained classifiers generalize to out-of-sample stimuli, allowing us to use naturalistic stimuli while containing the risk that some spurious stimulus feature is driving results—and allows us to compare EEG-based classification performance to classifiers based on potential confounds, such as visual features, on a common scale (out-of-sample prediction). By using stimuli from a real, online marketplace, we are able to decode not just individual choice behavior, but the aggregate choice behavior reflected in the market. To this end, we recorded EEG while subjects made funding decisions about project proposal pitches from crowdfunding site Kickstarter, as in Genevsky et al.^3^. Sliding the classifier across time like a searchlight, our analysis revealed the time course over which scalp-recorded neural activity is able to predict choice behavior with millisecond precision. As control analyses, we also attempt to predict market-level outcomes from the subjects’ behavioral data and from visual features of the stimuli. Results show that a seemingly sparse subset of the brain’s visual response predicts market-level outcomes, but a relatively larger set of neural patterns predict individual choice outcomes.

## Materials and methods

### Participants and ethics statement

18 (6 male and 12 female) individuals living in the Chicago area participated in the study, with ages ranging between 18 and 41 (M = 22.8, SD = 5.4, skew = 2.1); however, two subjects were subsequently removed from EEG analysis since too many trials were contaminated by artifacts (see “EEG preprocessing”). Participants were recruited through the University of Chicago’s human subject recruitment system, SONA Systems, and through word of mouth. All subjects gave written, informed consent before participating. All of the methods performed in the study were in accordance with relevant safety and ethics guidelines for human subjects research and were approved by the Social and Behavioral Sciences Institutional Review Board at the University of Chicago (IRB18 0587). This study was not a clinical trial.

### Stimuli

The first 36 stimuli were Kickstarter pitches generously provided by a member of the Knutson lab. These pitches were used as stimuli in Genevsky et al.^3^ and a full description of how these stimuli were selected is reported in Genevsky et al.^3^. Since an ERP paradigm requires substantially more trials than were used in Genevsky et al.’s^3^ fMRI work, we selected 55 more pitches from Kickstarter (while the projects were live) and formatted them identically to the original stimuli (resulting in 91 total stimuli). In keeping with Genevsky et al.’s^3^ stimuli, we selected only independent film project appeals, and in general we followed their stimulus selection procedure as closely as possible. While we cannot publicly share all stimuli used in the experiment, since the copyright for that content belongs to the original creators, we are happy to provide our formatted stimuli upon request.

### Experimental design

We used the same funding task used by Genevsky et al.^3^; experiment control scripts were provided by the Knutson lab for replication purposes, and were edited to send event markers for EEG data collection. Subjects were informed that, during EEG recording, they would be making funding decisions regarding a number of projects that had been posted on a crowdfunding website (www.kickstarter.com) and that they were being given a small endowment ($15) to donate to any of the projects if they so choose. This endowment was about three times larger than the one used by Genevsky et al.^3^ because we had subjects choose between about three times as many projects. They were told that, after the completion of the experiment, one of the projects would be selected by a random number generator, and that if they had selected to fund that project, then their fifteen dollars would be donated directly to the project on Kickstarter. Consequently, either the full $15 was donated to a single project or they took the full $15 home, as in Genevsky et al.^3^.

**Figure 1:**
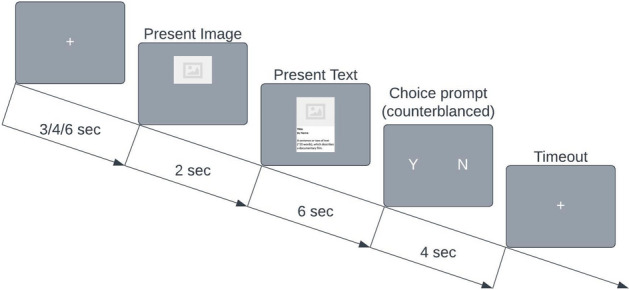
A graphical depiction of a single trial. Made using LucidChart (lucidchart.com).

Subjects were then shown pictures and text taken directly from the Kickstarter pages, without any information about others’ choices or about progress toward a funding goal as would be shown on the original Kickstarter page. Project appeals were presented in a random order for each subject. As in the original design^3^, subjects viewed an image from the project page (2 s) followed by both the image and the project’s short text description (1–2 sentences, usually around 20 words) displayed simultaneously for 6 s. Subsequently, subjects were given 4 s to answer whether they would like to fund the project using “Yes” or “No” buttons, which randomly switched between the sides of the screen between trials to prevent habitual motor planning. The choice prompt remained for a fixed duration regardless of the subjects’ response time, and no timer was shown to indicate how much time was remaining. After the choice period, subjects viewed a centrally presented fixation cross until the beginning of the next trial period (3, 4, or 6 s, chosen randomly for each trial). The time course of a single trial is visualized in Fig. 1. After the experimental session, placement of the electrodes on participants' heads was photographed using a geodesic positioning system with 11-mounted cameras to photogrammetrically determine the precise location of all 128 electrodes^8^. In total, 91 test stimuli were presented in randomized order using the MATLAB package PsychToolbox during EEG measurement. Overall, the task took less than 30 min.

### Electrophysiological data acquisition

Electroencephalographic (EEG) responses were recorded with an Electrical Geodesics, Inc. (EGI) GES 400 Amplifier with 129 electrodes (including the reference channel at vertex) on HCGSN 130 nets. Before beginning the recording, we ensured the impedances of all electrodes were below 50 kΩ. The GES 400 amp has a hardware sampling rate of 8 kHz, but it automatically applies an anti-aliasing filter and then decimates to 1 kHz before digitizing. No additional filtering was applied at acquisition. Trial tags were added using a photocell attached to the screen on which stimuli were displayed to measure the exact time of stimulus onset with sub-millisecond precision as calibrated on our equipment.

### Statistical analysis

We first assess the degree to which single trials of EEG predict (1) individual choices the subject makes and (2) the success of particular product pitches the subject is shown on the crowdfunding market. We take the same analytical approach to both prediction problems.

Our classification pipeline for each time point is as follows: the (preprocessed, see below) voltages measured at each EEG electrode are taken as the features for our classifier. Then, we train a logistic regression classifier fit using a generalized estimating equation (GEE) to predict behavioral outcomes (yes vs. no in the individual choice case, and funded vs. unfunded in the market-level outcome case) from these features. A generalized estimating equation is a procedure for estimating the parameters of a generalized linear model (GLM) while accounting for random effects^9^. In our case, this means we can fit a single classifier trained over all subjects. This has two advantages over the typical logistic regression classifiers used in much MVPA-based neuroimaging research: (1) since a single model is fit over all subjects, we can meaningfully visualize the model weights, and (2) GEE-fit GLMs still afford the statistical inference methods used in multiple regression, such as testing between nested models, which are precluded by the regularized regressions usually used for MVPA. Note, however, that while GEEs intuitively hold promise for improving the cross-subject generalization performance of MVPA classifiers compared to previously used methods, we do not specifically compare different methods here, nor is our cross-validation scheme designed to assess cross-subject generalization. We use the GEE implementation available in Python’s statsmodels package^10^, which we wrapped in a custom module consistent with the scikit-learn API^11^ for interoperability with Python-based MVPA frameworks. This tool may be useful for other researchers, and is available on our project GitHub (see *Data and Code Availability*).

The out-of-sample predictive performance of our classifier is evaluated using a 10 × 2 cross-validated t-test^12^. In this scheme, we compute the out-of-sample area under the receiver-operator characteristic curve (ROC-AUC or here just AUC) over 10 random half-half splits of the data (i.e. training the model on one half and computing ROC on the other. This procedure ensures that there is no overlap in stimuli between halves so we can be confident our results generalize to novel stimuli). Then, we compute a modified *t*-test on those out-of-sample AUC values. It is worth taking a moment to note the logic behind this choice of statistical test, especially given the lack of consensus in the neuroimaging field concerning how to evaluate statistical significance for cross-validation results. While it has become common practice to, for example, compute model performance metrics such as AUC and accuracy over many cross-validation (CV) splits (or folds) and then compare those metrics to their chance values with a *t*-test (where *n* is the number of splits used), the recycling of data across CV splits violates the independence assumption of a t-test. A simple *t*-test, while still seen in the literature, does not actually control the false positive rate in this setting^12^. In particular, it is easy to see that one could drive the standard error with which the *t-*statistic is computed arbitrarily low merely by increasing the number of cross-validation splits, since *n* is in the denominator of the standard error, which is inversely proportional to the *t*-statistic. To avoid this pitfall, Dietterich^12^ proposed a *k* × 2 *t*-test with a formula for the standard error which is not dependent on the number of splits in the cross-validation scheme, thus approximately controlling the false positive rate. More discussion of our statistical inference procedure, as well as the results of permutation tests of the same hypotheses (provided for comparison), can be found in Supplementary Information.

ROC-AUC was chosen as an evaluation metric for our classifier since it is less sensitive to class imbalances than is accuracy (and generally less prone to misleading/false positives), as it is independent of any particular decision threshold—rather, it is a non-parametric metric of how linearly separable our behavioral outcomes are given our predictors (electrode voltages).

We ran the full procedure described above on *each time point* in the epoch of interest relative to the onset of the image stimulus. This provides a separate classifier for each sample in our epoch (one unique classifier per millisecond). This allows us to assess when, relative to the onset of a stimulus, choice-related information is linearly decodable from scalp-recorded neural activity.

After performing this procedure on every time point for both contrasts (individual and market choice), it is necessary to correct for multiple statistical comparisons to control our false positive rate. We used All-Resolutions Inference (ARI)^13^ to identify temporal clusters in which decoding ROC-AUC was reliably above chance with a false discovery proportion (FDP) lower than 5%. (Technically, ARI can bound the true/false discovery proportion for arbitrary subsets of the data without specifying a target FDP, but we simply increase the clustering threshold until we reach FDP < 0.05). This procedure is theoretically related to the more common Benjamini–Hochberg procedure^14^ for controlling the false-discovery rate, but it accounts for the autocorrelation structure of the data in the selection of subsets (i.e. clusters). ARI allows for inference at the level of single data points within a cluster, unlike popular cluster-based permutation tests for M/EEG which fail to control the false positive rate for inferences about cluster extent^15^. Our implementation of ARI is available as a Python package, MNE-ARI^16^.

We additionally conduct two control analyses to account for potential alternative explanations of our decoding results. (1) One possibility is that the individual choices of our subjects happened to be sufficiently predictive of market-level outcomes that our aggregate-choice predictor was merely decoding individual behavior. To address this concern, we train a classifier (as used for neural decoding, a logistic regression fit using GEE) to predict market-level outcomes from just the subjects’ behavioral data. To ensure a fair comparison, we use the exact same cross validation split as used for the neural decoder. However, we also fit a logistic regression to the full sample to compute a more traditional significance metric for the sake of completeness. (2) Another possibility is that some simple visual feature present in the stimuli happens to predict funded from unfunded marker outcomes, and our neural decoder has just learned to decode that visual feature, and not aggregate choice itself. To this end, we input our stimuli into the popular image recognition model AlexNet^17^, and we extract the activations in the penultimate layer of the deep neural network as a vector representation of the visual features. Using these feature vectors, we ask whether any single visual feature can linearly separate funded and unfunded stimuli using a linear discriminant analysis compared to chance with another 10 × 2 cross-validated t-test.

Finally, since the GEE approach to training our decoder results in a single model per timepoint across all subjects, unlike more common approaches to MVPA that fit a separate classifier for each subject, we can interrogate the model weights to determine which features the decoding model deems important for classification. GEE-fit GLMs lend themselves to tests of nested models, as used with traditional multiple linear models. Thus, for each timepoint in which we are able to predict market outcomes from EEG above-chance (after correcting for multiple comparisons), we test the nested models that exclude subsets of electrodes as defined by the 10–20 system (excluding prefrontal electrodes, then frontal electrodes, then temporal, then parietal, then occipital) against the full model using a Wald’s test. Note that since we apply this procedure only on the models that we already know exceeded chance performance, this analysis is circular, leading to inflated false-positive rates^18^. Thus, this analysis should not, in any sense, be considered confirmatory; rather, results should be considered as potentially useful for hypothesis generation. Moreover, since inference is being done on families of predictive models, rather than directly on the data, results should be thought of as explaining the performance of the models themselves, not as explaining the EEG data. Given this strong limitation (i.e. the computed *p-*values do not carry their conventional meaning), we only report positive findings—that is, subsets of electrodes that the Wald’s test indicates excluding would hurt model performance—and we do not correct for multiple comparisons.

### EEG preprocessing

The EEG data were preprocessed in Python using the MNE-Python package^19^. First, common preprocessing operations were performed in adherence to the standardized PREP as implemented in the PyPREP package^20^. While the full PREP pipeline is described in detail by^21^, in summary, line noise is removed via multitaper, the signal is robustly re-referenced the average of all electrodes, bad channels are identified (by multiple criteria, including extreme amplitudes, lack of correlation with other channels, lack of predictability by neighboring channels, and unusual high frequency noise) and interpolated relative to this new reference, then a new “true” average reference is computed after channel interpolation. These steps are repeated iteratively until convergence, so that bad channel identification is not biased by the re-referencing computation, and the resulting average reference is a “true” average that excludes artifactual data from bad channels. (We only deviated from PREP in one instance, in which a bad channel was obviously missed by the pipeline; this was E44 from participant 109; interpolated channels for each subject are noted in their preprocessing reports.) After applying PREP, we bandpass filter the data from 0.3 to 50 Hz, and then we re-reference to create bipolar EOG channels from the electrodes below and above the eyes; in particular, we use electrodes E15–E126 and E8–E127 on the EGI net. We then broke the data into epochs ranging from 200 ms prior to stimulus onset to 800 ms after stimulus onset, holding off on any baseline correction until after applying ICA. We decompose the epoched data into 15 independent components (ICs), and we excluded components that are more correlated with the EOG channels than the others^22,23^. Specifically, we identified outliers by computing Pearson correlations between all ICs and both eye channels, z-score the correlations, and flag ICs with a z-score of greater than 1.96 or less than − 1.96. We then removed ICs flagged as containing eye artifact are then subtracted from the epoched data. (For transparency, scalp topographies of all removed ICs for all subjects are contained in the preprocessing reports in in the derivatives directory of the BIDS formatted dataset). Then, we baseline corrected the epochs by subtracting the average voltage in the 200 ms interval proceeding stimulus onset from each electrode in each epoch. We then identified artifactual trials as those that exceed a peak-to-peak amplitude threshold and drop them from the dataset. Optimal peak-to-peak rejection thresholds were determined on a per-subject basis using the Autoreject package^24^, which selects the threshold that minimizes the fivefold cross-validated mean-squared difference between the surviving trials and the average trial. (Individual rejection thresholds are recorded in each subjects’ preprocessing report available in the BIDS dataset). At this point, we excluded two subjects (110 and 114) from further analysis since their trial-averaged data did not contain a clearly visible evoked response. For transparency, their trial-averaged data is visualized in their preprocessing reports available in the BIDS dataset, as is the trial-averaged data of all subjects. Finally, the data were resampled to 100 Hz before applying the decoding analysis for computational efficiency, which should have resulted in no loss of information since the data had already been low-pass filtered at 50 Hz earlier in preprocessing.

## Results

**Figure 2:**
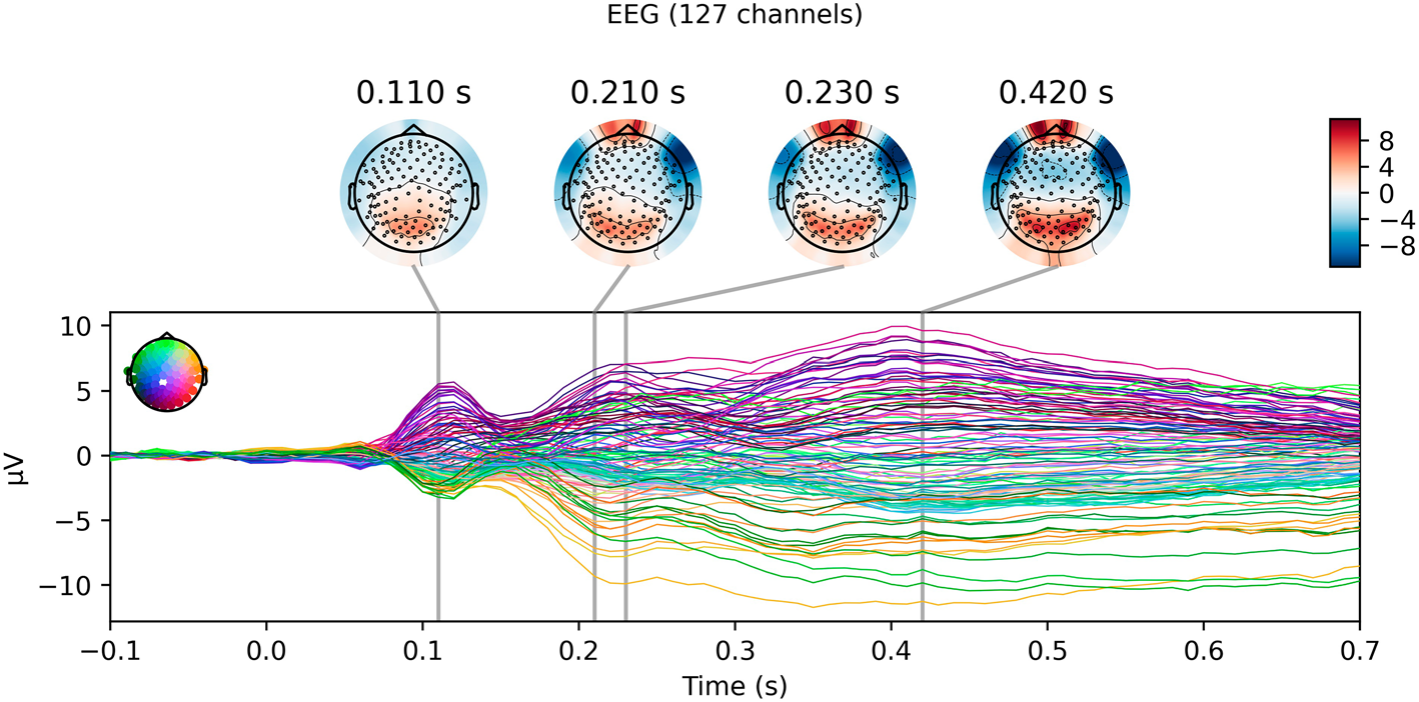
Grand average visually-evoked response to image stimuli. The EEG response to the presentation of the image stimuli (at 0.0 s), averaged over all clean trials within each subject and then averag across subjects.

A robust visually-evoked response was obtained in response to the image stimuli (see Fig. 2). Trial-by-trial variations in this response were able to predict both the behavior of the individual subjects and market-level outcomes for the project appeals from which the images were taken. We could not know with certainty which projects would be funded at the market level at the time the stimuli were gathered from Kickstarter; it ended up that 43 projects (stimuli) were funded and 48 were not funded. Our subjects, on average, our subjects chose to fund 21.4 (SD = 14.9) project appeals.

### EEG results

**Figure 3:**
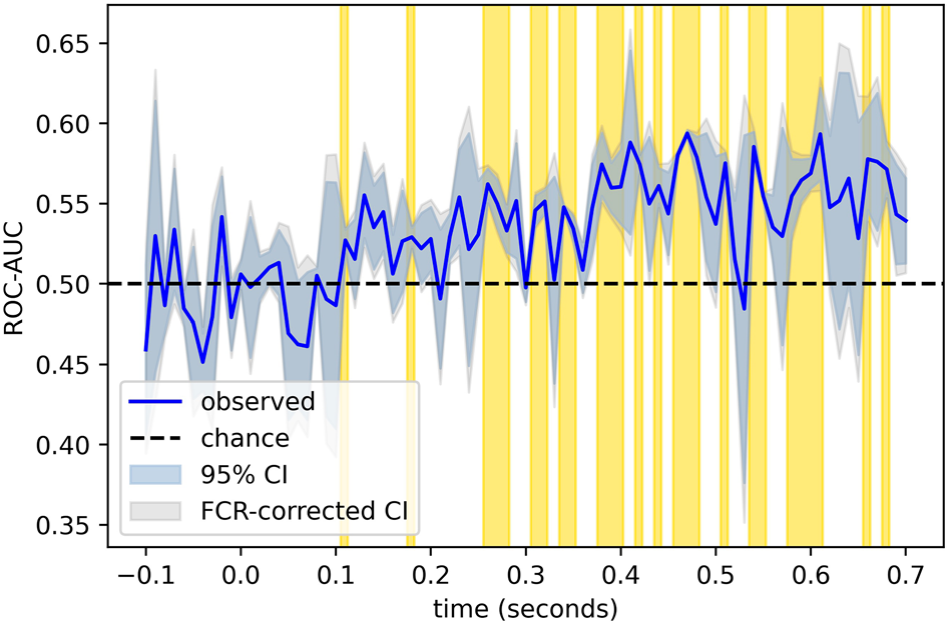
Cross-validated classification performances for individual choice outcomes. The area under the receiver-operator characteristic curve (ROC-AUC), a nonparametric measure of class separability, for our classifier’s ability to predict individual choice outcomes from single-trial EEG data in hold-out stimuli. 95% confidence bands are shown, and well as their corresponding false-coverage rate (FCR) adjusted confidence intervals using the method described by Rosenblatt and Benjamini^25^. Regions in which ROC-AUC exceeds chance performance with a false-discovery of less than five percent are highlighted in yellow.

**Figure 4:**
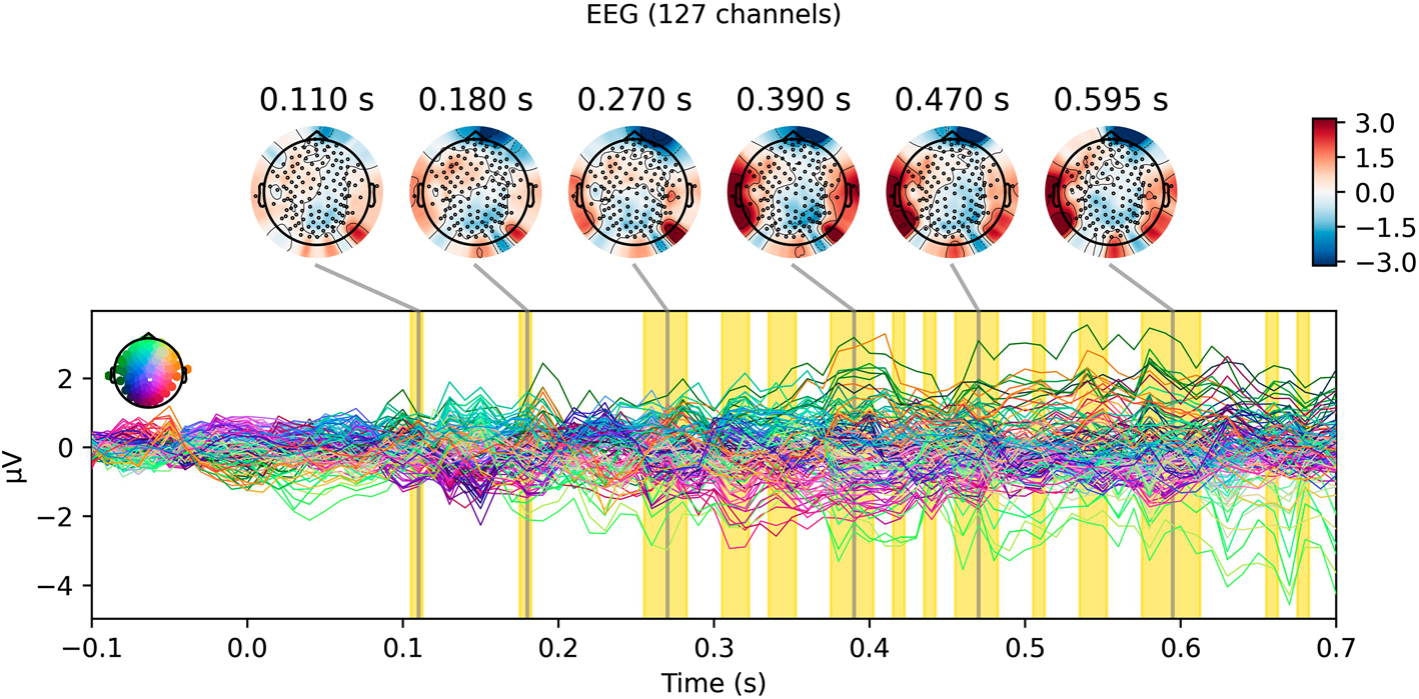
Patterns that predict individual choice. The patterns of scalp-recorded activity that our model uses to differentiate trials in which subjects choose to fund from those in which they do not, reconstructed from the model weights using the method described by Haufe et al.^26^. This can be interpreted similarly to a difference wave for “yes” trials minus “no” trials. Clusters in which classification performance exceeds chance are highlighted in yellow.

The single-trial EEG data was able to predict individual choice behavior (see Figs. 3 and 4) on new (out-of-training-sample) stimuli with an AUC that reached up to about 0.584, corresponding to a probability of 58.4% that the classifier would rate an arbitrary “yes” trial as higher than an arbitrary “no” trial. Classification performance first exceeded chance (*p* = 6.4e−6) beginning 110 ms after image onset, during the N1 cortical response to the image, and it nominally trended upward throughout the epoch, achieving peak performance between 460 and 480 ms after the onset of the stimulus image. Please note, though, that the permutation test reported in Supplementary Information results in somewhat different time windows being labelled as significant; most notably, the windows between 300 and 400 ms drop out. However, the onset of above-chance decoding (at 110 ms) remains the same, and robust decoding performance subsequent to 400 ms is still observed. Thus, the critical features for our interpretation of the data (see “Discussion”) are consistent across multiple statistical approaches.

**Figure 5:**
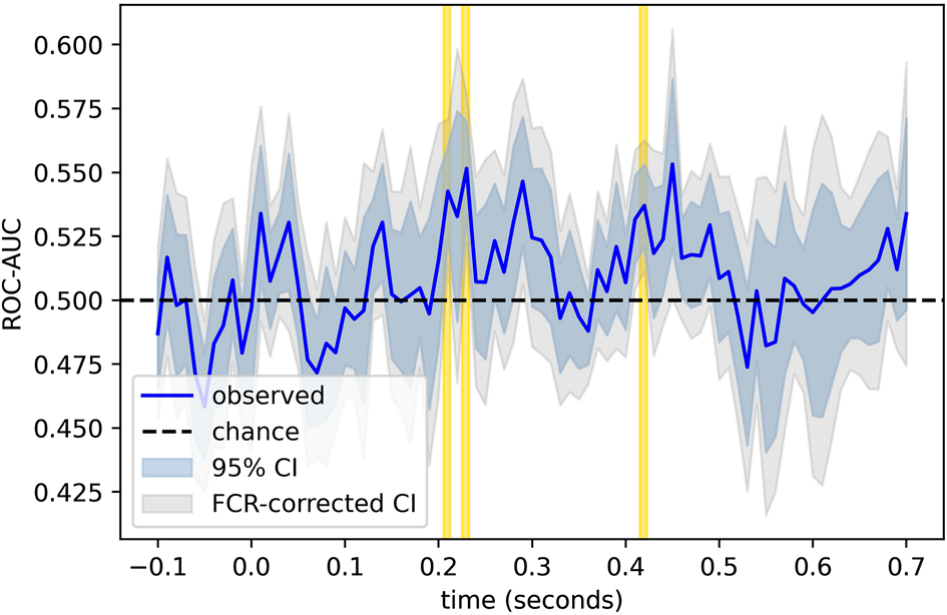
Cross-validated classification performances for aggregate choice outcomes. The area under the receiver-operator characteristic curve (ROC-AUC), a nonparametric measure of class separability, for our classifier’s ability to predict market outcomes from single-trial EEG data in hold-out stimuli.

**Figure 6:**
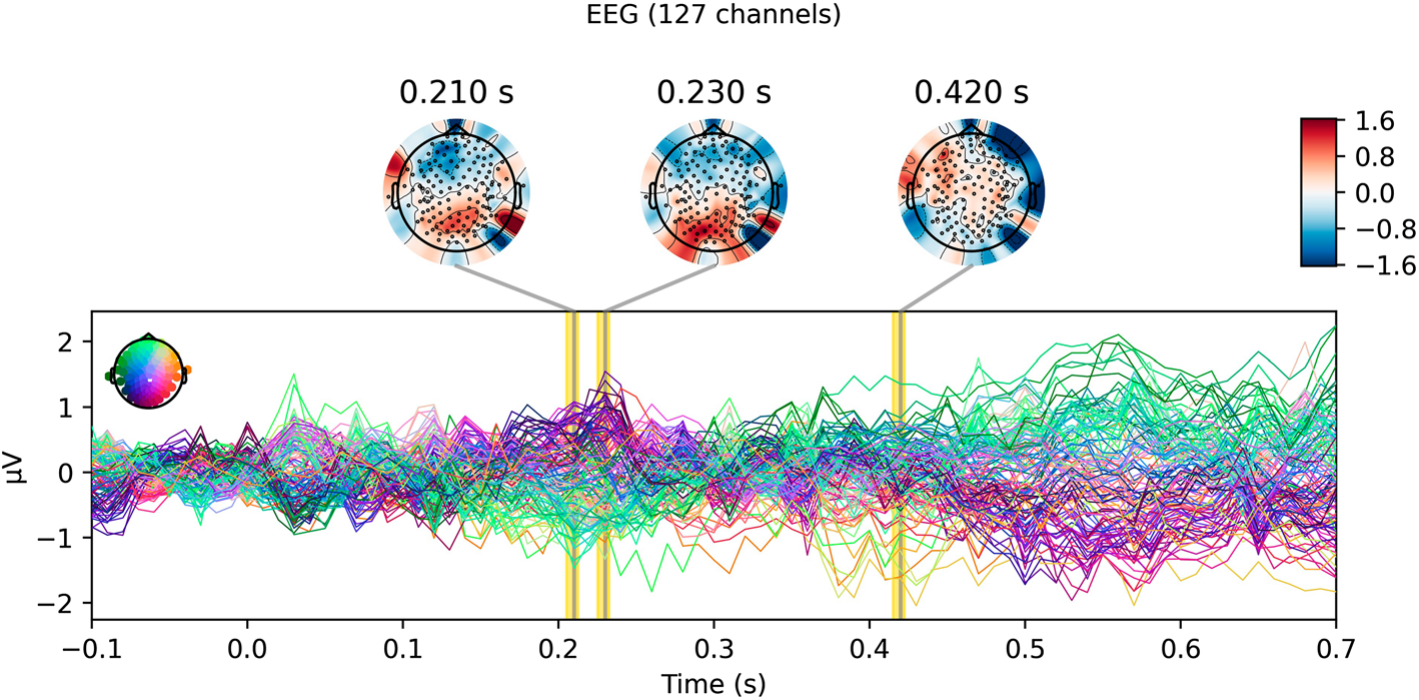
Patterns that predict market outcomes. The patterns of scalp-recorded activity that our model uses to differentiate trials in which the product pitch presented to the subject ultimately reached their funding threshold on Kickstarter from those in which they did not. This can be interpreted similarly to a difference wave for “funded” trials minus “unfunded” trials. Clusters in which classification performance exceeds chance are highlighted in yellow.

The EEG also predicted whether crowdfunding appeals (i.e. the individual stimuli) would ultimately reach their funding threshold on Kickstarter (see Figs. 5 and 6). Thus, single trial EEG during choice behavior can also predict market-level outcomes, or the aggregate behavior of a group. The first subset of EEG activity that predicted market behavior above-chance (*p* = 0.0004) began somewhat later, at around 210 ms after image onset and never preceded the stimulus. Peak predictive performance occurred 230 ms after image onset, reaching an AUC of 0.552, and a later window of above-chance predictive performance followed at 420 ms (*p* = 0.0005).

### Control analysis I: behavioral data

Cross-validated AUC for predicting market outcomes from subjects’ individual choices (using the same cross-validation splits as the results visualized in Fig. 4) was 0.5 (95% CI [0.45, 0.55), which fails to reach significance (*t*(10) = 0.17, *p* = 0.44). When we forgo cross-validation and simply fit a logistic regression via GEE within-sample, we obtain a model coefficient of 0.151 (95% CI [− 0.078, 0.381]), failing to detect a relationship between our subjects’ individual choices and market outcomes even within-sample (*z* = 1.294, *p* = 0.196). It is, then, unlikely that the relationship between individual and aggregate choice can account for the decoding results above.

### Control analysis II: visual features

When predicting market-outcomes from vector representations of the image stimuli (taken from the penultimate layer of AlexNet, see “Materials and methods”) using the first Linear Discriminant dimension, we obtain an ROC-AUC of 0.49 (95% CI [0.39, 0.59]), failing to reach statistical significance (*t*(10) = − 0.241, *p* = 0.593). Thus, no single visual feature that we might be able to decode from the EEG would seem to be able to account for our ability to decode market outcomes.

### Exploratory analysis: features that account for classification of aggregate choice

Please note, as mentioned in “Materials and methods”, that the results of this exploratory analysis are in no sense confirmatory, and they are not corrected for multiple comparisons. The following should be interpreted only as potentially valuable for hypothesis generation.

While the pattern of activity used to predict market outcomes 210 ms after stimulus onset appears to nominally emphasize both frontal and occipital electrodes (see Fig. 6), the only subset of electrodes that a Wald’s test suggest would hurt classification performance if excluded is the prefrontal electrodes (*p* = 0.007). At 230 ms after target onset, Wald’s test instead highlights the importance of parietal electrodes for predicting aggregate choice (*p* = 0.03). In the later window that predicts aggregate choice, at 430 ms after stimulus onset, Wald’s test indicates that both prefrontal (*p* = 0.04) and central (*p* = 0.02) electrodes are important for successful market outcome classification. No other subset of electrodes (defined by 10–20 the anterior-to-posterior 10–20 system nomenclature) results in a significant Wald’s test at α = 0.05 when excluded from the decoding models.

## Discussion

It is now well known that sensory systems play an important role in perceptual decision-making, representing decision parameters (e.g. risk and reward) rather than merely stimulus attributes (e.g. color, motion)^4–6^. Whether such findings generalize from perceptual decisions with fixed reward-payoff structures to value-based decisions in which the rewards are determined by the decision-maker remains an open question in the literature^27^. Indeed, the striatum, medial prefrontal cortex, and other brain structures not typically associated with sensory perception are thought to encode the representation of subjective value^28,29^. While previous research has shown that early (< 250 ms) visually evoked neural activity correlate with subsequent choice outcomes^30–32^, the (out-of-sample) predictive power of these correlates have not been previously characterized. Previously, researchers have found that the magnitude of the EEG visual response indexes individuals’ consumer preference in the N1-P2 (< 250 ms) time window^30–32^ as well as during the late slow-wave of the visual response^32^. Similarly, our multivariate analysis approach highlights early predictors of choice behavior, beginning very shortly (~ 110 ms) after stimulus onset, with greater decoding performance attained during the late slow-wave.

Of particular interest, here, is the neural activity that predicts the aggregate (i.e. market-level) behavior related to the visual stimuli. Activity in the P2 time window, around and following 200 ms, predicts market-level outcomes. Neural activity in the P2 time window following a visual stimulus is usually characterized by EEG researchers as resulting from feedforward volleys of activity from the optic nerve, ascending through primary and secondary visual cortices^30–32^. However, evidence from human and nonhuman studies suggests that a widespread system of sensory, parietal, and prefrontal areas come online very early (as soon as ~ 30 ms) in the visually evoked response^33^ which could support rapid cognitive processing. An exploratory analysis of our model weights suggest that prefrontal electrodes preferentially drive predictive performance in this time interval, though this does not imply a particular neural source.

Importantly, while patterns that predict the choices of individual subjects are commonplace after 250 ms, the signals we find predict market outcomes are relatively sparse. Of course, we must be careful not to interpret a null result in those times where we did not find above-chance predictive performance; the lack of evidence could be due to insufficient predictive power. (And indeed, in the logical extreme in which the sample size approaches the population size, individual and aggregate choice become congruent.) However, it is interesting to note that topographies predicting individual choice qualitatively differ from those predicting aggregate choice, even showing opposite polarity in the prefrontal electrodes we find are important for aggregate prediction in the early (near 210 ms) window, though individual choice prediction is not significant at that time. Since scalp EEG measures an aggregate of neural processes occurring in parallel across the brain, only some of which Genevsky et al. (2017) would argue scale to predict market outcomes from small study samples, then using both individual and aggregate outcomes as regression targets for MVPA may facilitate the dissociation of temporally overlapping processes.

Taken together, one might conjecture that an early cortical network as described by Foxe and Simpson^33^ may be involved in the initiation of decision-making processes. This inference would contrast—at least on the surface—with Genevsky et al.’s^3^ finding that reward-anticipatory nucleus accumbens activation, but not cortical activation, predicts market outcomes during decision-making. However, it is worth noting that studies using electrodes implanted in the nucleus accumbens as part of deep-brain-stimulation treatment have reported top-down-directed synchrony from medial frontal cortex to nucleus accumbens during reward anticipation^34^. From this, one possibility is that we observe a time-resolved cortical correlate of the subcortical predictors described by Genevsky et al.^3^. This possibility precludes us from inferring that the observed predictive patterns originate solely from the visual system, without the involvement of the traditional reward system. Moreover, the ~ 230 and ~ 420 ms windows further implicate parietal and central electrodes in predicting market outcomes, suggesting new avenues for future investigation.

Importantly, the evoked response examined here is only to the picture associated with the product pitch and not the text description subjects (or Kickstarter patrons) read only later. Given this early EEG response predicts market success, one interpretation is that these perceptual responses to the image may often carry weight in real-world funding decisions independently of the persuasive text of the pitch. This is consistent with the kind of argument that some researchers have made about intuitive, implicit decision making^35,36^ as compared with more deliberative, explicit decisions^37^. The implicit/explicit process dichotomy has proven a useful, albeit simplistic, frame for understanding behavior in the cognitive and behavioral economics literature. However, the neural basis of this dichotomy is not self-evident. This finding, therefore, suggests a new direction for future research: fast, intuitive decision processes may be grounded in perceptual systems.

## Conclusion

Our results are consistent with a partial scaling account of choice behavior^1^, in which a subset of the neural processes underlying decision-making are able to predict the aggregate behavior of the population (in this case, of a market) even when the behavior of sampled individuals deviates from the aggregate. Importantly, however, the specifics of our findings differ from the partial scaling account proposed by Knutson and Genevsky^1^ due to the temporal precision afforded by EEG. While previous work has found that subcortical affect-related activity scales to predict market outcomes^3^, our work finds that cortical activity during early- to middle-latency visual processing also scales to predict market-level outcomes from the neural activity of individuals. While it could be construed as surprising that activity during visual processing would predict value-based decisions at all, much less the behavior of markets, we posit that early selection processes (e.g. visual attention) are rather well-suited to drive behavioral consistency across individuals. After all, early selection in principle impacts all subsequent cognitive processes; in contrast, later cognitive and neural processes are constrained to act on the output of earlier processing steps. In this vein, the scaling of individual affective responses to predict market behavior observed by^1,3^ could reflect that fact that affective responses to visual stimuli tend to have short latencies, rather than a property specific to affective processes per se. We can return, then, to our original question: What neural processes reinforce consistent behavior across individuals, such that one can extrapolate from a small sample of individuals to predict the aggregate behavior of a large population? Our results would suggest that behavior which is highly consistent within a population (requiring smaller samples for such an extrapolation to be successful) is often driven by early processes—perceptual, attentional, or affective—rather than by later (necessarily idiosyncratic) abstract cognition.

### Supplementary Information

Supplementary Information.

## Data Availability

All raw EEG and behavioral data, as well as the preprocessed EEG data and quality-check reports output by our preprocessing pipeline, is available on OpenNeuro (https://doi.org/10.18112/openneuro.ds004284.v1.0.0) and is formatted according to the Brain Imaging Data Standard^38,39^. Similarly, the package specifications needed to reproduce our computational environment, as well as the Python code used for EEG preprocessing, neural decoding, control analyses, and visualization of results (i.e. the code for producing Figs. 2, 3, 4, 5, 6) is available on GitHub (https://github.com/john-veillette/eeg-neuroforecasting). While stimuli are not publicly available (since we do not own the content used in the stimuli), we are happy to privately share them with other researchers upon request.
