# Author Correction: Single-trial visually evoked potentials predict both individual choice and market outcomes

**DOI:** 10.1038/s41598-024-53532-z

**Published:** 2024-02-06

**Authors:** John P. Veillette, Shannon L. M. Heald, Benjamin Wittenbrink, Katherine S. Reis, Howard C. Nusbaum

**Affiliations:** Department of Psychology, University of Chicago, Chicago, 60637 USA

Correction to: *Scientific Reports* 10.1038/s41598-023-41613-4, published online 01 September 2023

Katherine S. Reis was omitted from the author list in the original version of this Article.

The Author Contributions section now reads:

“CRediT (https://credit.niso.org). J.V.: Conceptualization, Methodology, Software, Validation, Visualization, Formal analysis, Investigation, Data Curation, Writing – Original Draft, Visualization, Project administration. S.H.: Conceptualization, Methodology, Writing—Review & Editing, Supervision, Project Administration. B.W.: Methodology, Data Curation, Writing—Review & Editing. K.R.: Data Curation, Writing—Review & Editing. H.N.: Conceptualization, Methodology, Resources, Writing—Review & Editing, Supervision, Funding acquisition.”

The original Article has been corrected.

