# Supplementary material for: Single-trial visually evoked potentials predict both individual choice and market outcomes
Source: Sci Rep. 2023 Sep 1;13:14340. doi: 10.1038/s41598-023-41613-4 (PMC10474019; doi:10.1038/s41598-023-41613-4)
Supplement: Supplementary file 1 — Supplementary Information. [file 41598_2023_41613_MOESM1_ESM.pdf]

Supplementary Information

1. Supplementary Methods

1.1. Significance Testing for Cross-Validation Performances

The area under the receiver-operator curve (ROC-AUC) is a non-parametric measure of class separation, which follows a known probability distribution; specifically, it is directly proportional to the generalized *U*-statistic used in the non-parametric Mann-Whitney *U*-test, which follows a normal distribution with known mean under the null hypothesis (Mason et al., 2002). If the ROC-AUC values we measured were independent measurements, we could have used this known distribution to compute a *p*-value directly, which constitutes an accepted statistical test for comparing receiver-operator curves to each other or to chance (DeLong et al., 1988). However, since our cross-validated ROC-AUCs are *not* independent, as the training and test sets used to compute them overlap across repeated cross-validation folds (Bengio and Grandvalet, 2003), we instead compared them to chance (ROC-AUC = 0.5) in the main text using a version of the *t*-test that explicitly accounts for (1) co-dependence between cross-validated measurements and (2) variability in the test statistic due to random cross-validation splits (Dietterich, 1998). Since the other assumptions of a *t*-test are handily met by a sample of generalized *U*-statistics, this procedure can be expected to conservatively control the false-positive rate. Since we use a parametric *t*-test on a non-parametrically computed *U*-statistic, one might call our significance testing procedure *semi-parametric*.

In the larger machine learning literature, the problem of significance testing for comparing the cross-validation performance of classifiers is well-studied, and approaches such as ours that leverage known distributional information are often preferred (Nadeau and Bengio, 1998; Bouckaert and Frank, 2004), as they offer exact *p*-values and improved computational expediency over randomization-based methods. However, the multi-variate pattern analysis literature tends to favor permutation or Monte-Carlo simulation approaches to significance testing (e.g. Bae and Luck, 2018) to account for biases that may occur in the random cross-validation splits (though this is mainly a problem when the test statistic is sensitive to class imbalances, such as accuracy). As mentioned above, our testing procedure is relatively insensitive to these anticipated biases, but we nonetheless present permutation test results here to demonstrate consistency with the semi-parametric approach we take in the main text.

1.2 Permutation Tests

For our permutation tests, we compute the same corrected, cross-validated *t*-statistic as in the main text (Dietterich, 1998) at each time point across 10,000 permutation of the test labels, using the same cross-validation scheme across permutations so that any bias induced by the cross-validation splits would be reflected in the permutation null distribution. Since we use a one-tailed test, as only above-chance decoding performance is interpretable, the *p*-value at each time-point is computed as one minus the percentile rank of the observed *t*-statistic in the permutation null distribution (i.e. the test is significant at a level of 0.05 if the *t*-statistic is at or exceeds the 95th percentile of the permutation null, or falls outside of the middle $90^{\%}$ of the distribution).

However, it is necessary to correct for multiple comparisons. In this vein, we use the *t*-max procedure to strongly control the family-wise error rate (Nichols and Holmes, 2002), in which a null distribution is constructed by taking the largest *t*-statistic (across all individual tests / time points) seen on each permutation – that is, the max-*t* statistic. Each individual test is then compared not to its own null distribution, but to the null distribution of the max-*t* statistic; the multiple-comparisons corrected rejection threshold, then, is the 95th percentile of the max-*t*'s null distribution.

We additionally visualize the observed ROC curves for market-level predictions against 1,000 permutation ROC curves at select time points (namely, those times at which market-level prediction is significant after correcting for multiple comparisons).

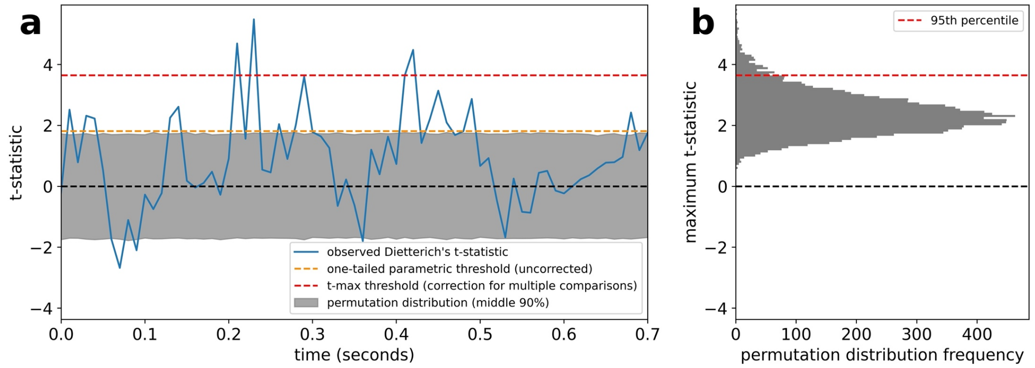

Supplementary Figure 1: Permutation test results for prediction of aggregate choice outcomes. (a) Observed value of Dietterich's corrected *t*-statistic plotted against the middle 90% of the permutation distribution of that *t*-statistic. The parametric rejection threshold (before correcting for multiple comparisons for Dietterich's *t*-test is shown for comparison (orange), and it aligns closely with the rejection threshold derived from the permutation distribution. The *t*-max corrected rejection threshold, which controls the familywise error rate, is shown in red. **(b)** Histogram of the permutation distribution of the max-*t* statistic; the 95th percentile of this distribution constitutes the new rejection threshold, after correcting for multiple comparisons.

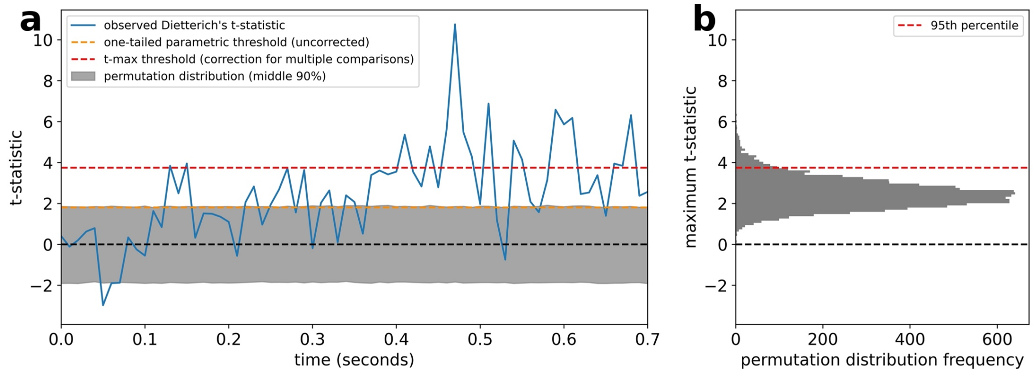

Supplementary Figure 2: Permutation test results for prediction of individual choice outcomes. Contents are the same as in Supplementary Figure 1.

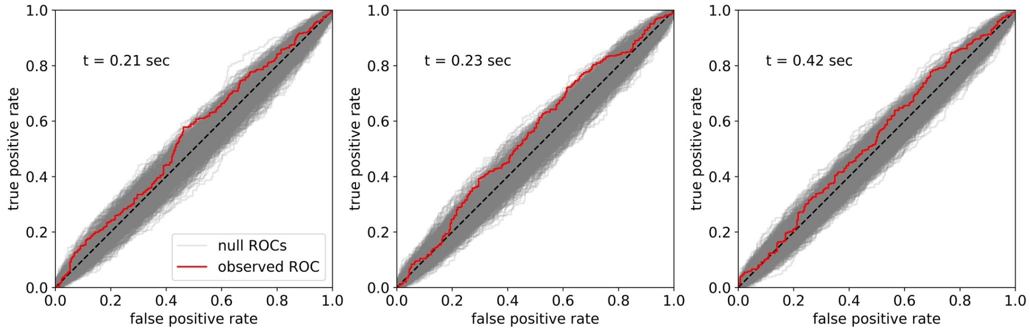

Supplementary Figure 3: Receiver-operator curves for prediction of aggregate choice outcomes at selected time points. The observed ROC (red) for the first cross-validation split is shown against 1,000 ROC curves computed with shuffled class labels (grey).

2. Supplementary Discussion

Results of the permutation procedure largely mirror the results of the semi-parametric approach we use in the main text. In particular, the permutation test rejects the null hypothesis (chance classification) at an identical set of time points for market-level prediction after correcting for multiple comparisons (see Supplementary Figure 1).

The exact times at which the null is rejected for individual choice prediction do vary slightly from the times reported in the main text. In particular, the second significant interval moves up in time, and those between 300 and 400 ms appear to drop out. However, the features critical to our interpretation – the timing of the earliest window and the largely sustained above-chance decoding performance following 400 ms – remain the same.

Moreover, in both cases, we find that the permutation null distribution of Dietterich's corrected *t*-statistic is nicely centered around zero, and the parametric rejection threshold aligns closely with that derived from the permutation distribution. This suggests that Dietterich's *t*-test indeed controls the false-positive rate, and the semi-parametric approach used in the main text is valid.

Supplementary References

Bae, G. Y., & Luck, S. J. (2018). Dissociable decoding of spatial attention and working memory from EEG oscillations and sustained potentials. *Journal of Neuroscience*, *38*(2), 409-422.

Bengio, Y., & Grandvalet, Y. (2003). No unbiased estimator of the variance of k-fold cross-validation. *Advances in Neural Information Processing Systems*, *16*.

Bouckaert, R. R., & Frank, E. (2004, May). Evaluating the replicability of significance tests for comparing learning algorithms. In *PAKDD* (Vol. 3056, pp. 3-12).

DeLong, E. R., DeLong, D. M., & Clarke-Pearson, D. L. (1988). Comparing the areas under two or more correlated receiver operating characteristic curves: a nonparametric approach. *Biometrics*, 837-845.

Dietterich, T. G. (1998). Approximate statistical tests for comparing supervised classification learning algorithms. *Neural computation*, *10*(7), 1895-1923.

Mann, H. B., & Whitney, D. R. (1947). On a test of whether one of two random variables is stochastically larger than the other. *The annals of mathematical statistics*, 50-60.

Mason, S. J., & Graham, N. E. (2002). Areas beneath the relative operating characteristics (ROC) and relative operating levels (ROL) curves: Statistical significance and interpretation. *Quarterly Journal of the Royal Meteorological Society: A journal of the atmospheric sciences, applied meteorology and physical oceanography*, *128*(584), 2145-2166.

Nadeau, C., & Bengio, Y. (1999). Inference for the generalization error. *Advances in neural information processing systems*, *12*.

Nichols, T. E., & Holmes, A. P. (2002). Nonparametric permutation tests for functional neuroimaging: a primer with examples. *Human brain mapping*, *15*(1), 1-25.
